# Macrophage migration inhibitory factor inhibition as a novel therapeutic approach against triple-negative breast cancer

**DOI:** 10.1038/s41419-020-02992-y

**Published:** 2020-09-17

**Authors:** Manish Charan, Subhadip Das, Sanjay Mishra, Nabanita Chatterjee, Sanjay Varikuti, Kirti Kaul, Swati Misri, Dinesh K. Ahirwar, Abhay R. Satoskar, Ramesh K. Ganju

**Affiliations:** 1 Department of Pathology, Ohio State University, Columbus, OH 43210 USA; 2 Comprehensive Cancer Center, Ohio State University, Columbus, OH 43210 USA

**Keywords:** Breast cancer, Apoptosis

## Abstract

Triple-negative breast cancer (TNBC), defined as loss of estrogen, progesterone, and Her2 receptors, is a subtype of highly aggressive breast cancer with worse prognosis and poor survival rate. Macrophage migration inhibitory factor (MIF) is a pleiotropic pro-inflammatory cytokine aberrantly expressed in many solid tumors and known to promote tumor progression and metastasis. However, its role in TNBC progression and metastasis is unexplored. Here we have shown that in TNBC patients, MIF expression was significantly enriched in the tumor compared to adjacent normal tissue. Using publically available patient datasets, we showed that MIF overexpression correlates with worse survival in TNBC compared to other hormonal status. Orthotopic implantation of TNBC cells into MIF knockout mice showed reduced tumor growth compared to wild-type mice. In addition, we have shown that MIF downregulation inhibits TNBC growth and progression in a syngeneic mouse model. We further showed that CPSI-1306, a small-molecule MIF inhibitor, inhibits the growth of TNBC cells in vitro. Mechanistic studies revealed that CPSI-1306 induces intrinsic apoptosis by alteration in mitochondrial membrane potential, cytochrome *c* (Cyt *c*) release, and activation of different caspases. In addition, CPSI-1306 inhibits the activation of cell survival and proliferation-related molecules. CPSI-1306 treatment also reduced the tumor growth and metastasis in orthotopic mouse models of mammary carcinoma. CPSI-1306 treatment of tumor-bearing mice significantly inhibited TNBC growth and pulmonary metastasis in a dose-dependent manner. Histological analysis of xenograft tumors revealed a higher number of apoptotic cells in CPSI-1306-treated tumors compared to vehicle controls. Our studies, for the first time, show that MIF overexpression in TNBC enhances growth and metastasis. Taken together, our results indicate that using small molecular weight MIF inhibitors could be a promising strategy to inhibit TNBC progression and metastasis.

## Introduction

Despite extensive efforts to develop effective therapies against breast cancer, it remains the leading cause of mortality of women around the world^1^. Based on molecular and immunohistochemical parameters, breast cancer can be classified into different subtypes, including highly aggressive triple-negative breast cancer (TNBC). The major receptors utilized to develop hormonal therapies against breast cancer, including human epidermal growth factor 2 receptors (HER2), estrogen receptors (ER), and progesterone receptors (PR), are lost in TNBC, making it difficult to treat with conventional therapies^2^. TNBC constitutes 15–20% of invasive breast cancers. In TNBC, the development of early resistance to chemotherapies and widespread metastasis leads to shorter overall and relapse-free survival compared to other breast cancer subtypes^3,4^. Therefore, there is an utmost need to develop novel therapies against highly aggressive and metastatic TNBC.

TNBC with an inflammatory phenotype is associated with a poorer prognosis. However, the exact role of inflammatory molecules in promoting TNBC is yet not defined. Macrophage migratory inhibitory factor (MIF) is an inflammatory molecule, initially discovered as an inhibitor of macrophage random migration^5,6^. MIF can drive its oncogenic signaling via both autocrine and paracrine manners. MIF is known to bind with the CD74 receptor to activate several inflammatory and survival pathways such as MAPK and PI3K/Akt^7^, but the exact molecular mechanism is not fully understood. Interestingly, CD74 or MIF blockade reduced the aggressiveness of invasive breast cancer cells^8^.

In recent years, several reports have shown that MIF is overexpressed in many cancers, including breast, colorectal, lung, prostate, and head and neck cancers. Its overexpression is believed to play a pro-tumor function by regulating both cell proliferation and tumor aggressiveness^9–11^. Furthermore, higher expression of MIF has been reported as a prognostic factor for various human malignancies^12–14^. Also, high levels of circulating MIF is associated with poor prognosis in breast cancers^8^. The critical biological activities required for tumor initiation and progression, including angiogenesis, cell proliferation, and apoptosis, are regulated by MIF. Therefore, targeting MIF could be a promising strategy to target aggressive breast cancers, including TNBC. A small molecular inhibitor of MIF, CPSI-1306, has been shown to block the biological activity of MIF both in vitro and in vivo. However, the preclinical efficacy of CPSI-1306 against TNBC is yet not investigated.

Here, we demonstrate that MIF plays an important role in TNBC growth and metastasis. Furthermore, MIF inhibitor CPSI-1306 significantly inhibits TNBC cell viability by inducing the mitochondrial apoptosis pathway. CPSI-1306 treatment in preclinical mouse models reduced TNBC tumor burden and distant pulmonary metastasis.

## Materials and methods

### Reagents

Dulbecco’s modified Eagle’s medium (DMEM), fetal bovine serum (FBS), penicillin and streptomycin (PS) antibiotic, trypsin, and ethylene diamine tetraacetic acid were obtained from Gibco BRL (Grand Island, NY, USA). Tissue culture plastic wares were obtained from NUNC (Roskilde, Denmark). DAPI was procured from Invitrogen (CL, USA). Human recombinant MIF was purchased from Sigma.

### Cell culture and treatments

Cells were cultured in DMEM supplemented with 10% FBS and 1% antibiotic (PS) at 37 °C in a humidified atmosphere under 5% CO_2_. Human TNBC cell lines MDA-MB-468 and MDA-MB-231 were purchased from American Type Culture Collection (ATCC). MVT-1 cell line was a kind gift from Dr. Johnson (Georgetown University Medical Centre, Washington and DC). All cell lines were routinely checked for mycoplasma contamination and verified based on cell morphology before all experiments are performed.

For MIF stimulation, CD74-downregulated MDA-MB-231 MIF knockout (KO) cells were incubated in DMEM medium containing 0.2% FBS and 100 ng/ml of recombinant MIF at 37 °C for 3 h. Changes in protein phosphorylation were analyzed by western blot (WB).

### Cell viability assay

Cell viability assay was performed using MTT dye. Briefly, 1 × 10^3^ cells were seeded per well in 100 μl of medium in 96-well plates (Nunc, Roskilde, Denmark). Cells were treated with different concentrations of CPSI-1306 (Tocris, MN, USA) for 48 h. After the incubation, medium was aspirated and MTT solution was added to each well and incubated for 3 h and cells were further suspended in MTT solvent. Absorbance was measured at 590 nm. The absorbance correlates linearly to the number of living cells in culture, and the IC_50_ value was calculated. In addition, cell viability was also assessed using a Prestoblue dye (Thermo).

### Plasmids, lentivirus production, and siRNAs

The clustered regularly interspaced short palindromic repeats (CRISPR)/Cas9 system targeting human MIF was purchased from Applied Biological Materials (ABM Inc.) and MIF was knocked out in TNBC cell line as per the manufacturer’s instructions. The shRNA constructs targeting MIF were cloned into pLKO.1 hygro vector (a gift from Bob Weinberg Addgene plasmid 24150) and lentiviral particles were generated as described in Addgene’s pLKO.1 protocol. siRNAs targeting MIF and CD74 were purchased from Dharmacon and transfected with Lipofectamine 2000 (Invitrogen) into breast cancer cell lines. shRNA sequences are given in Supplementary Table 1.

### Cell fractionation and flow cytometry

Cytoplasmic, nuclear and mitochondrial fractions were separated by using a cell fractionation kit (Cell Signaling Technologies, cat # 9038).

For cell-cycle analysis, MDA-MB-468 and MVT-1 cells were treated with vehicle control or CPSI-1306 for 48 h and cell-cycle distribution was assessed through flow cytometry (LSR II BD) by staining the DNA with propidium iodide (PI) (BD Biosciences) as per the manufacturer’s instructions. 1 × 10^4^ cell events were recorded for each sample and analyzed using FlowJo software.

*Cell death*: Percent apoptosis was determined by using FITC-labeled Annexin-V and PI kit (BD Biosciences). Briefly, after treatment with CPSI-1306, MDA-MB-468 and MVT-1 cells were stained with Annexin-V-FITC and PI according to the manufacturer’s instructions. 1 × 10^4^ cells were recorded for determining the percentage of live, apoptotic, and necrotic cells using flow cytometry (LSR II BD) and analyzed using FlowJo software.

*CD74 expression*: For the detection of membrane-bound CD74, TNBC cells were incubated with CD74 polyclonal antibody (CST) for 30 min followed by four washes with phosphate-buffered saline (PBS) and incubated with AF-594-conjugated goat anti-rabbit IgG (Invitrogen) for 1 h in dark. Samples were recorded on LSRFortessa and analyzed using FlowJo software. An anti-rabbit IgG1 antibody was used as an isotype control.

### Immunoblotting

Immunoblotting was performed as described in ref. ^15^. Briefly, cell lysates were separated on NuPAGE 4–12% gradient precast gels (Invitrogen), transferred onto 0.45 μm nitrocellulose membranes (BioRad), and probed with the appropriate dilution of specific primary antibodies (Supplementary Table 3). HRP-conjugated secondary goat anti-mouse IgG or anti-rabbit IgG (CST) were used and proteins were developed using a chemiluminescent substrate (Millipore). Glyceraldehyde 3-phosphate dehydrogenase (CST) and β-actin (CST) were used as loading controls. WB images were quantified using BioRad Quantity one software.

### Measurement of mitochondrial membrane potential and reactive oxygen species (ROS) generation

The mitochondrial membrane potential was evaluated using the fluorescent probe TMRM dye (Invitrogen) as per the manufacturer’s instructions. The membrane potential was measured on a flow cytometer (LSR II BD). ROS production was measured by H2DCFDA (Invitrogen) as per the manufacturer’s protocol.

### Terminal deoxynucleotidyl transferase dUTP nick end labeling (TUNEL) assay

Paraffin-embedded tumor tissue sections (4 μm thick) were deparaffinized and rehydrated using a descending concentration of alcohol. Tissue slides were further processed for TUNEL assay (Molecular Probes) following the manufacturer’s protocol.

### Migration and wound-healing assay

Briefly, 1 × 10^5^ cells were seeded in serum-free medium (200 μl) on an 8-μm filter insert (Corning) and 2% FBS (200 μl) was used in the bottom chamber as a chemoattractant. Migration was set for 8 h. and migrated cells were stained using the Diff kwik staining kit as per the manufacturer’s instructions. Colonies were counted on a bright field microscope. For wound-healing assay, 3 × 10^5^ cells/well were seeded in a 24-well plate. After the formation of a complete monolayer, cells were scratched using a yellow tip. After scratching, cells were washed once with PBS and incubated for 24 h. Images at different time points (0 and 24 h) were taken on a light microscope.

### Colony-forming assay

1 × 10^3^ cells were seeded in each well of a six-well plate in DMEM supplied with 10% FBS. The next day the media were changed to DMEM with 3% FBS and incubated for 6 days with vehicle control or CPSI-1306. Cells were fixed, stained, and colonies were counted^16^.

### Immunofluorescence assay

Immunofluorescence (IFA) on mammalian cells was performed as described in ref. ^17^. Briefly, TNBC cells were cultured on chambered slides and fixed with methanol for 30 min at 4 °C. Cells were permeabilized with 0.1% Triton X-100 in PBS for 5 min at room temperature. After five washes with PBS, cells were blocked in 5% bovine serum albumin (BSA) for 30 min, samples were incubated overnight with primary antibody against human Cyt *c* (dilution,1:200, CST, #4272) and apoptosis-inducing factor (AIF) (dilution,1:200, CST, cat no 5318) at 4 °C. Unbound primary antibody was removed by washing four times with PBS and samples were incubated with Alexa-Fluor-conjugated secondary antibodies (Life Technologies) for 2 h at room temperature. After washing five times with PBS, slides were mounted with DAPI and analyzed under a confocal microscope (Zeiss LSM 700).

### Tissue microarray (TMA)

TMA slides containing paraffin-embedded TNBC patient tissues were processed at the Pathology Core Facility and Tissue Archives Human Tissue Resource Network at Ohio State University. TMA includes a total of 100 samples with 61 TNBC tumor sections and 39 adjacent normal samples. Immunohistochemistry (IHC) on these slides was performed using MIF antibody (dilution, 1:1000, Sigma) and analyzed by using an IHC profiler^18^.

### Immunohistochemistry

IHC was performed as described in ref. ^19^. Briefly, 4-μm-thick tissue sections were deparaffinized with xylene, rehydrated with descending alcohol series followed by antigen retrieval in citrate buffer. Sections were stained using the Vectastain Elite ABC kit and ImmPACT DAB Peroxidase Substrate following the manufacturer’s method (Vector Laboratories). Primary antibodies against anti-human Ki67 (dilution, 1:100; Dako, MIB-1) and anti-human CD31 (dilution, 1:1000; Dako, clone JC70A) were used.

IFA alike IHC on paraffin-embedded tissues was carried out with some modifications. Briefly, after antigen retrieval, sections were blocked using 5% BSA for 1 h at room temperature. Sections were then stained for primary antibodies against human Ki67 (dilution, 1:100, Thermo, 14-5698-82), vascular endothelial growth factor (VEGF) (dilution, 1:100, Thermo, MA5-12184), AIF (dilution, 1:100, CST, cat no 5318), intercellular adhesion molecule (ICAM) (dilution, 1:100, Thermo, MA5407 and CD31 (Santa Cruz at 1:100 dilution). Alexa-Fluor-conjugated (AF-488 and AF-594) secondary antibodies (Life Technologies) were used for detection. Sections were mounted by Vectashield mounting media containing DAPI (Vector Laboratories, Inc.). Images were visualized on a confocal microscope (Zeiss, LSM 700).

### Animal studies

All experiments were approved by the Institutional Animal Care and Use Committee of the Ohio State University and animals were housed as per University Laboratory Animal Resources guidelines. Female FVB, C57BL/6, and NOD/SCID/IL-2gamma (NSG) mice were purchased from Charles River Laboratories Inc. MVT-1 or MDA-MB-231 (5 × 10^5^ cells) were implanted orthotopically into the fourth mammary gland of WT FVB (*N* = 5) and NSG (*N* = 5) female mice, respectively. When tumors became palpable, mice were randomized and treated orally with CPSI-1306 (10 or 20 mg/kg; three times in a week) for 4 weeks. Also, MDA-MB-231 scramble (scr) and MIF knockdown (sh1) cells were injected into the mammary gland. Tumor volume was measured using an external digital caliper. Tumor volume was calculated using the formula *V* = LA × SA^2^ × 0.5 where LA is largest and SA is the smallest superficial diameter^20^. After completion of treatment, mice were euthanized; tumors and lungs were harvested and fixed using formaldehyde. Lung nodules were counted after fixation. Tumors were analyzed for weight, proliferation, and apoptosis markers. Similarly, E0771 cells were also injected into the fourth mammary gland of WT (*n* = 10) and MIF KO mice (*n* = 10) and observed for tumor growth.

### Statistical analysis

For the in vitro experiments, three replicates per group were used. Statistics were performed using Prism software (GraphPad Software Inc., San Diego, CA, USA). Unpaired Student’s *t*-tests were used for comparing two groups, and one-way ANOVA was used for comparing more than two groups followed by Tukey’s post hoc test. For Curtis and TCGA dataset, ***P < 0.001 cut-off value was used for calculating statistical significance by using *t*-test and ANOVA^21,22^. Statistical significance was noted in the figures as *P < 0.05, **P < 0.01, ***P < 0.001 and ^#^non-significant.

## Results

### MIF expression in breast cancer patients

**Fig. 1:**
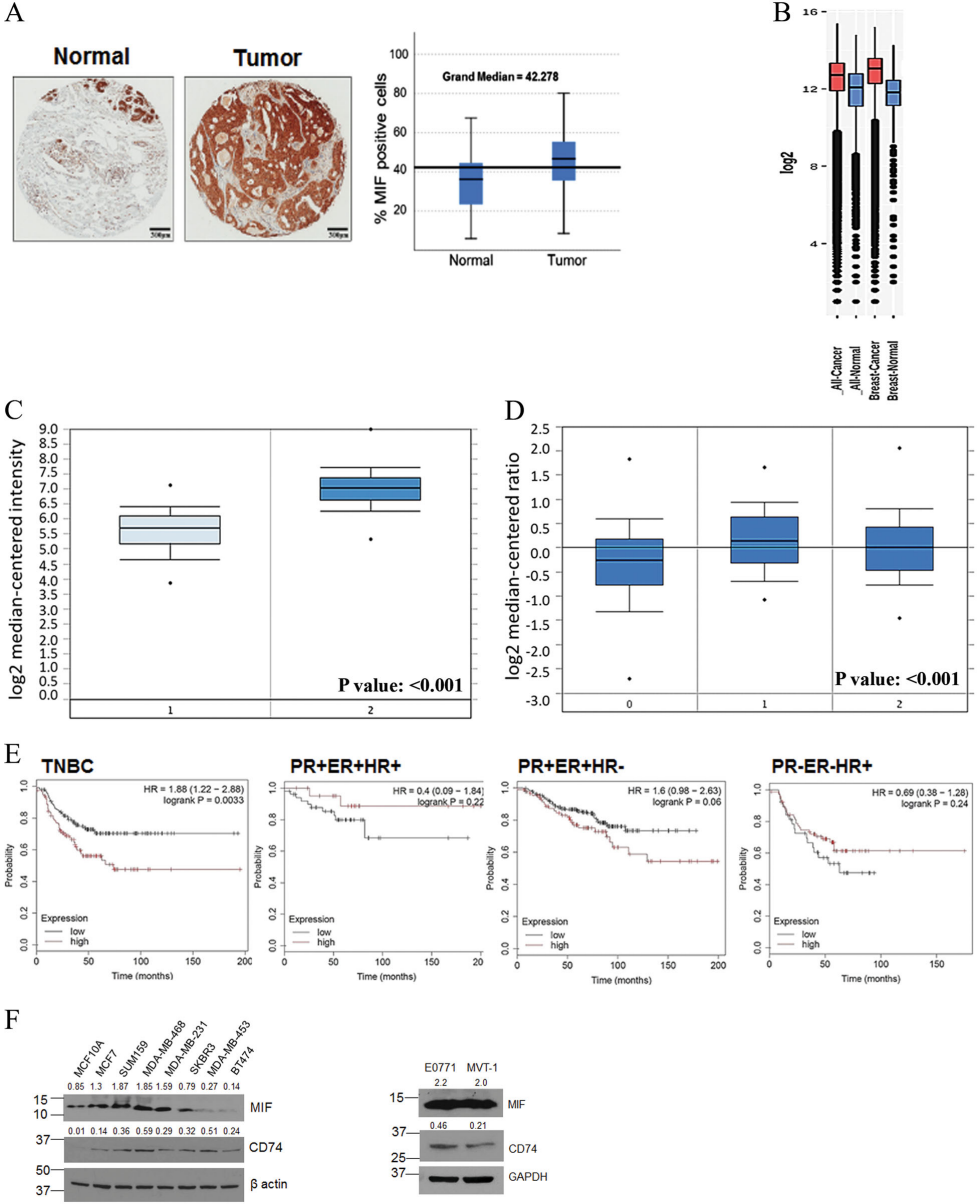
Analysis of MIF expression in human breast cancer patient samples. **a** Representative picture of MIF expression in human breast cancer patient TMA that contained (*n* = 61) TNBC and (*n* = 39) normal adjacent samples. The non-parametric independent-sample median test was used to calculate *P* value and statistical significance. The median value of percent MIF-positive cells was significantly (0.0001) higher in TNBC than the normal adjacent tissue. **b** Using the GENT2-gene expression database, significantly higher expression of MIF (*P* < 0.001) in all cancer and breast cancer patients compared to normal subjects was observed. **c** MIF expression is significantly enriched in^2^ invasive ductal breast carcinoma (*n* = 1556) compared to^1^ normal breast (*n* = 144) in Curtis breast dataset. **d** MIF is significantly overexpressed in TNBC patients compared to other breast cancer subtypes using TCGA dataset. [0 = no value or unidentified hormonal status (*n* = 297), 1 = HER2/ER/PR negative (*n* = 46), 2 = other breast cancer biomarkers (*n* = 250)]. **e** KM-plotter survival analysis show that MIF mRNA expression (Affy ID: 217871_s_at) correlates with poor overall survival only in TNBC patients (*n* = 255) compared to other breast cancer subtypes. ER^+^PR^+^HR^+^ (*n* = 73), ER^+^PR^+^HR^−^ (*n* = 339), and ER^−^PR^−^HR^+^ (*n* = 115). **f** Expression of MIF and CD74 in human and murine TNBC cell lines. Cell lysates from MCF10A, MCF7, SUM-159, MDA-MB-468, MDA-MB-231, SKBR3, MDA-MB-453, BT474, E0771, and MVT-1 cells were analyzed for MIF and CD74 expression by WB. Western blots were quantified arbitrarily and values were added on top of each image. GAPDH: loading control.

Prognostic significance of MIF expression in breast cancer was assessed by a comprehensive analysis of MIF expression in human TNBC samples using TMAs that contained 100 patient samples with 61 TNBC tumor samples and 39 adjacent normal samples by immunohistochemistry. We detected a significantly increased expression of MIF in TNBC patient samples compared to adjacent normal controls (Fig. 1a). We further evaluated the expression of MIF in breast cancer patients using publically available datasets. We observed a higher expression of MIF in all cancers including breast cancer compared to normal subjects using a freely available GENT2-gene expression database^23^ (Fig. 1b). In addition, elevated MIF expression was observed in invasive ductal breast carcinoma patients compared to healthy individuals in Curtis breast cancer dataset (Fig. 1c). Furthermore, using TCGA dataset, we found that MIF expression was elevated in TNBC patient samples compared to other hormonal breast cancer subtypes (Fig. 1d**)**. Importantly, using KM-plotter survival analysis, we found that higher expression of MIF significantly correlates with worse overall survival in TNBC subjects exclusively, compared to other hormonal breast cancer subtypes (Fig. 1e). Finally, we detected an aberrant protein expression of MIF in various human and murine TNBC cell lines compared to cells from other breast cancer subtypes (Fig. 1f). These data suggest that MIF is overexpressed in TNBC and its expression correlates with worse survival probability.

### MIF depletion reduces tumor growth and metastasis of TNBC cells

**Fig. 2:**
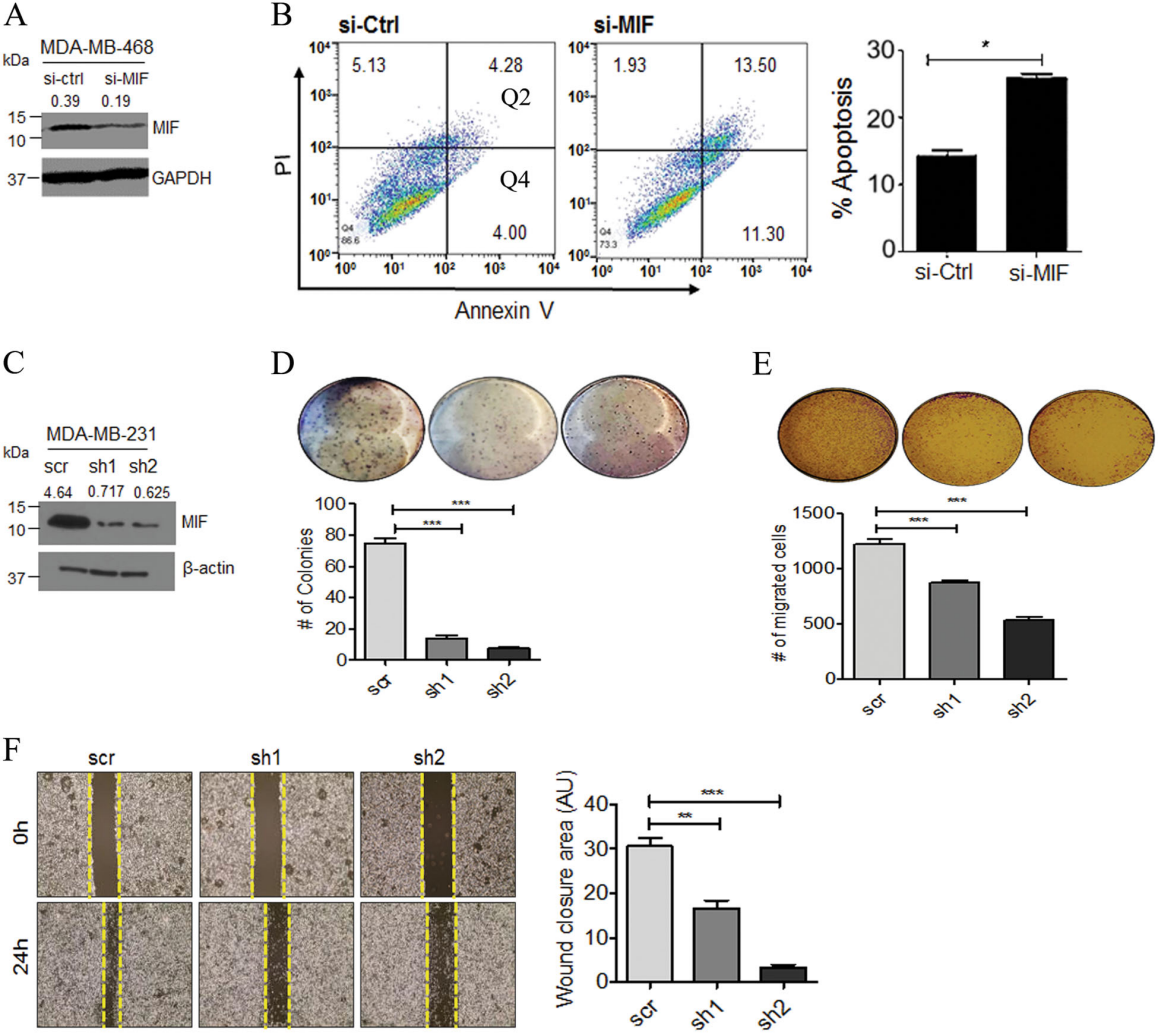
MIF knockdown inhibits TNBC cell proliferation and migration. **a** Western blot showing successful MIF knockdown in MDA-MB-468 cells by using an MIF targeting siRNA. **b** Flow cytometry was performed on control and MIF-specific siRNA-treated MDA-MB-468 cells to determine apoptosis by using Annexin-V and PI staining. Representative flow cytometry scatter plot of the gated cell population was presented. Quadrant Q4 and Q2 represent early and late apoptosis and percent apoptosis was quantified in bar graphs. **c** Western blot reveals stable downregulation of MIF using a lentiviral vector-mediated knockdown in MDA-MB-231 cells. **d**–**f** Colony formation, migration, and wound-healing assays were performed using stable MIF knockdown clones (sh1 and sh2). All loading controls provided beneath each protein of interest, belong to the same gel, which was used for western blot (WB) analysis. WB were quantified arbitrarily and values were added on top of each image. Bar graphs represent the quantification of the events. The data presented here is the mean ± SEM of triplicate experiments (*P < 0.05, **P < 0.01, ***P < 0.001).

To elucidate the role of MIF in cell survival and cellular migration, MIF was knocked down by using MIF-specific siRNA (si-MIF) in MDA-MB-468 cells (Fig. 2a). Knockdown of MIF in MDA-MB-468 significantly increased the apoptosis as compared to vector control cells (Fig. 2b). Next, to interrogate the role of MIF in vitro and in vivo, stable MIF knockdown clones were generated by using two independent shRNA targeting MIF in MDA-MB-231. MDA-MB-231 cells were used for MIF knockdown as they are reported to form mammary fat-pad xenograft tumors with a 100% metastasis rate in NOD/SCID/IL2rγ^null^ (NSG) immunocompromised mice^24^. MIF knockdown in MDA-MB-231 cells was confirmed by WB (Fig. 2c). MIF knockdown in MDA-MB-231 cells caused a significant reduction in colony-forming ability (Fig. 2d). In the transwell migration assay, the number of cells migrated through the porous membrane was also significantly low in MIF knockdown cells compared to scramble control cells (Fig. 2e). Also, MIF knockdown cells show reduced wound-healing capacity compared to scramble control cells (Fig. 2f). These results suggest that MIF plays an important role in regulating TNBC growth and migration.

**Fig. 3:**
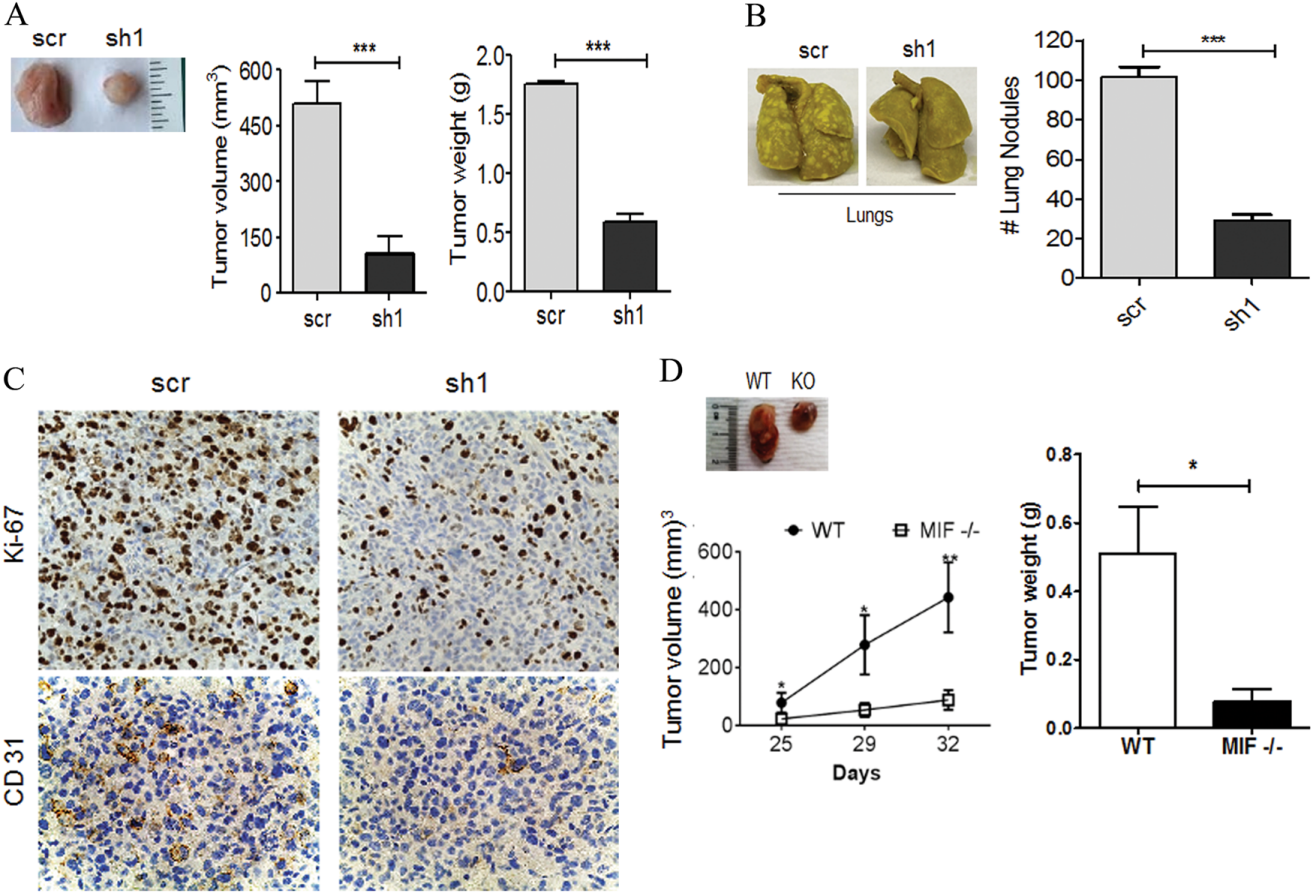
MIF downregulation reduces TNBC growth and metastasis. **a** MIF knockdown and scramble control MDA-MB-231 cells (5 × 10^5^) were injected into the mammary gland of NSG mice (*n* = 5). Representative photograph of tumors harvested from mice bearing MIF control (scr) and MIF knockdown (sh1) tumors. On endpoint arrival, tumors were excised from mice and tumor volume (left) and tumor weight (right) were measured. **b** Representative photographs of lungs derived from mice bearing MIF control and MIF knockdown tumors. The bar graph represents the number of metastatic colonies in the lungs. **c** MIF control and knockdown derived tumor tissues were subjected to IHC staining for Ki67 and CD31 expression. **d** E0771 cells (5 × 10^5^) were injected into the mammary gland of MIF knockout (KO) or wild-type (WT) C57BL/6 mice (*n* = 20). Representative photograph of tumors. Tumor volume (left) and tumor weight (right) were measured. The data reported here is the mean ± SEM of triplicate experiments (*P < 0.05, **P < 0.01, ***P < 0.001).

To examine the role of MIF in promoting tumor growth and metastasis, we used MIF expressing (scr) and MIF depleted (MIFsh1) MDA-MB-231 cells in vivo. These cells were injected into the mammary fat pad of NOD/SCID/IL2rγ^null^ (NSG) mice. Both groups of mice developed tumors and on the arrival of endpoints, MIF-depleted MIF-sh1 xenograft tumors showed significantly reduced tumor volume and tumor weight compared to scr xenograft tumors (Fig. 3a). Moreover, significantly reduced lung metastases were observed in mice bearing MIF-sh1 tumors than scr tumor bearing group (Fig. 3b). Our results demonstrate that MDA-MB-231-MIF-sh1 tumors showed reduced expression of Ki67+ (active proliferation) and CD31+ (blood vessel formation) as compared to scramble control groups (Fig. 3c). Host-derived MIF has been shown to promote tumor growth^25^. Using an orthotopic mouse model of MIF KO, we observed a significantly reduced tumor growth in MIF KO mice compared to wild-type (WT) mice (Fig. 3d) emphasizing the indispensability of host secreted MIF for tumor growth and progression. In conclusion, these findings establish an important role of MIF in tumor growth, progression, and metastasis of TNBC cells.

### CPSI-1306, a small-molecule MIF inhibitor has potent anti-proliferative activity against TNBC cells

**Fig. 4:**
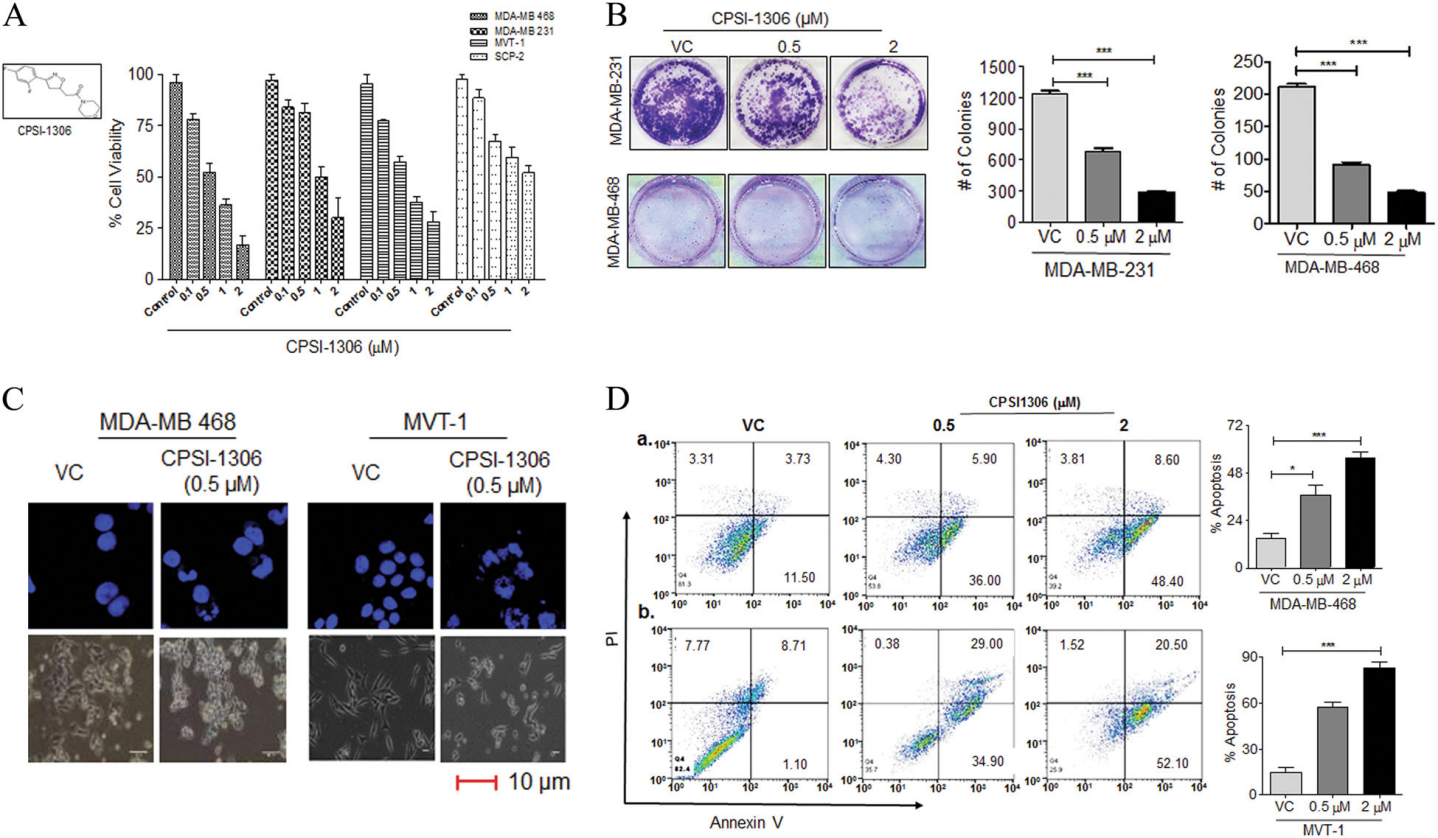
Growth inhibitory effect of CPSI-1306 on TNBC cell lines. **a** Cells were treated with vehicle control (VC) or CPSI-1306 for 48 h and cell viability was assessed using MTT assay. **b** Colony-forming ability was tested by treating the cells with vehicle control or CPSI-1306 for 6 days followed by staining and colony count. Representative images are shown and bar graphs (right) show quantification of colonies. **c** CPSI-1306 induced morphological and nuclear changes in MDA-MB-468 and MVT-1 cells were observed under light and fluorescence microscopy. **d** CPSI-1306-induced apoptosis was estimated using flow cytometry with Annexin-V and PI. Representative flow cytometry scatter plot of the gated cell population is presented. Percent apoptosis was quantified in adjacent bar graphs. The data reported here are the mean ± SEM of triplicate experiments (*P < 0.05, **P < 0.01, ***P < 0.001). Scale bars: 10 μm.

Since we observed that MIF is overexpressed in TNBC and enhance breast cancer growth and migration in vitro and in vivo, we evaluated the anticancer potency of a small-molecule chemical inhibitor of MIF to inhibit the proliferation and progression of TNBC. We used CPSI-1306, which has been reported to inhibit MIF activity^26^. A panel of human (MDA-MB-231, MDA-MB-468, SCP-2) and mouse (MVT-1) TNBC cells were treated with CPSI-1306 and cell viability was estimated by MTT assay. CPSI-1306 significantly reduced cell viability in nearly all TNBC cell lines (Fig. 4a). The IC_50_ values of CPSI-1306 for various cell lines are presented in Supplementary Table 2. We selected 0.5 and 2 μM concentrations of CPSI-1306 based on IC_50_ data for further experiments. In addition, we tested the MIF-specific cytotoxic activity of CPSI-1306 by treating it with MDA-MB-231-scr and MDA-MB-231-MIF-KO cells. We observed that MIF KO cells did not respond significantly compared to scramble control cells (Supplementary Fig. 1).

Next, we examined the effect of CPSI-1306 on cell survival and proliferation using colony formation assay. We found that CPSI-1306 significantly inhibited the colony-forming ability of TNBC (Fig. 4b). To evaluate whether the reduced ability of cells to form colonies is due to the induction of apoptosis, both the human and murine TNBC cells, MDA-MB-468 and MVT-1, were treated with CPSI-1306 and showed a classic morphology of apoptotic cells, including condensed and fragmented nuclei compared to the intact nucleus of vehicle-treated cells (Fig. 4c). In addition, CPSI-1306 treatment led induction of apoptosis in human and murine TNBC cells was analyzed by flow cytometry using FITC-labeled Annexin-V and PI in human and murine TNBC cells. A significantly increased percentage of apoptotic cells were observed in CPSI-1306-treated TNBC cells compared to the vehicle controls (Fig. 4d).

These studies demonstrate the potential of CPSI-1306 in inducing apoptosis in TNBC cells. The coupling of cell-cycle progression and apoptosis is required to maintain tissue homeostasis. This process is disrupted in various disease conditions, including cancers. To assess the effect CPSI-1306 on cell-cycle progression in TNBC cells, cell-cycle kinetics were studied using PI staining. The treatment of CPSI-1306 in MDA-MB-468 and MVT-1 cells caused a G2/M phase arrest in a dose-dependent manner (Supplementary Fig. 2). In brief, these results suggest that CPSI-1306 treatment restricts cellular growth and leads to apoptosis in TNBC cells.

### MIF/CD74 interaction regulates cell survival in TNBC cells

**Fig. 5:**
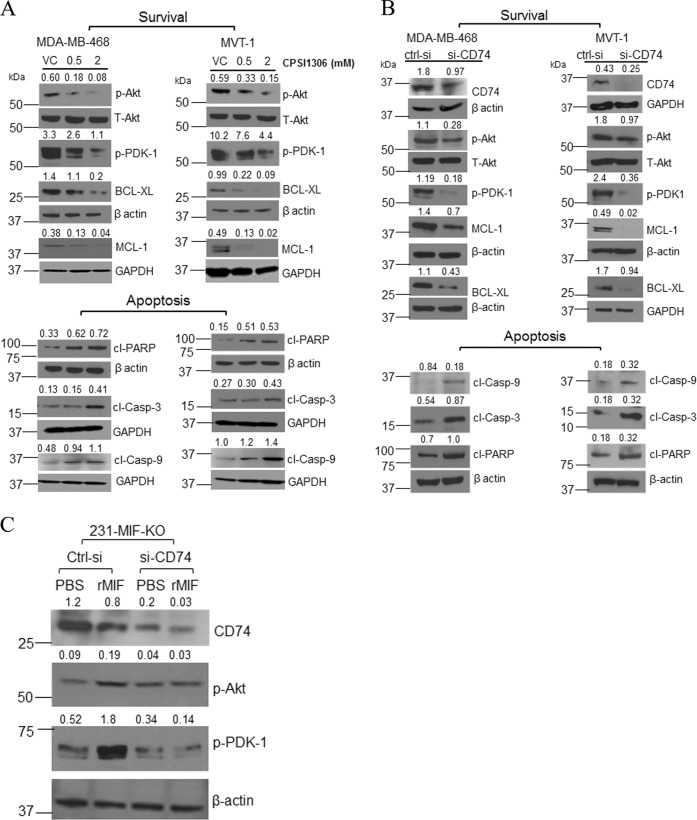
CPSI-1306 treatment activates pro-apoptotic proteins and suppresses cell survival markers. **a** Phosphorylation and expression status of various pro-apoptotic and cell survival markers in cell lysates from TNBC cells (MDA-MB-231 and MVT-1) treated with vehicle control (VC) or CPSI-1306 were analyzed by WB. **b** CD74 knockdown associated changes in activation and expression of various proteins in MDA-MB-468 and MVT-1 cell lysates were analyzed by WB. **c** CD74 was knockdown in MIF knockout MDA-MB-231 cells, these cells were exogenously supplemented with recombinant human MIF protein and analyzed for phosphorylation of p-Akt and p-PDK-1 by WB, and all proteins were normalized against β-actin. All loading controls provided beneath each protein of interest, belong to the same gel, which was used for western blot analysis. The data reported here is the mean ± SEM of three independent experiments. WB was quantified arbitrarily and values were added on top of each image.

MIF-mediated activation of ERK and phosphoinositide-3-kinase (PI3K)/Akt signaling has been shown to promote tumorigenesis and inhibit the programmed cell death^6,27,28^. To analyze whether pharmacological inhibition of MIF can induce apoptosis in TNBC cells, we treated MDA-MB-468 or MVT-1 cells with CPSI-1306. The treatment of CPSI-1306 in TNBC cells reduced the activation of cell survival proteins p-AKT (Ser-473) and p-PDK-1 (Ser-241) in a dose-dependent manner (Fig. 5a). However, CPSI-1306 treatment enhanced the expression of pro-apoptotic protein such as cleaved caspase-9 in TNBC cells (Fig. 5a). Also, CPSI-1306 treatment induced the expression of late apoptotic protein markers: cleaved caspase-3 and cleaved PARP (Fig. 5a). On the contrary, CPSI-1306 treatment suppressed the expression of anti-apoptotic mitochondrial membrane proteins: Bcl-XL and MCL-1 (Fig. 5a). These observations strongly suggest that CPSI-1306 mediates its antitumorigenic effects by inhibiting cell survival pathways and stimulate programmed cell death in TNBC cells.

CD74 is a multifunctional cell surface protein and a receptor of MIF. Therefore, we postulated that autocrine MIF/CD74 signaling might play a crucial role in the survival of TNBC cells. Firstly, we analyzed the expression of CD74 in TNBC cell lines and observed a basal expression of CD74 (Fig. 1). Next, to decipher the role of CD74, CD74 was knockdown in MDA-MB-468 and MVT-1 cells (Fig. 5b), and it markedly reduced cell proliferation, and induced apoptosis (Fig. 5b). In addition, CD74 downregulation induced apoptosis and CD74 cell surface expression was confirmed by flow cytometry (Supplementary Fig. 3A, 3B). Likewise, in MIF knockdown, similar downregulation of cell survival proteins and anti-apoptotic proteins was observed in CD74-knockdown cells (Fig. 5b). CD74 knockdown caused a reduction in the phosphorylation of cell survival proteins like PDK-1 and Akt (Fig. 5b**)**. Since the PI3K/AKT pathway is a key cell survival signaling pathway, we also evaluated the expression of the anti-apoptotic proteins such as Bcl-XL and MCL-1 (Fig. 5b). Additionally, expression of early and late apoptotic proteins, including cleaved PARP, cleaved caspase-9 and 3 were also found increased (Fig. 5b). These results suggest that cell-secreted MIF binds to CD74 in an autocrine fashion and regulate the phosphorylation of PDK-1 and Akt and expression of Bcl-XL, MCL-1, cleaved PARP, and cleaved caspase-9 and -3.

Furthermore, to establish the essentiality MIF/CD74 signaling axis, we generated MIF-KO MDA-MB-231 cells using CRISPR/Cas9 (Supplementary Fig. 4). These cells also showed reduced phosphorylation of Akt, one of MIF’s downstream targets (Supplementary Fig. 4). CD74 was downregulated in these MIF KO cells using a CD74-siRNA and exogenously supplemented with functional recombinant human MIF protein (100 ng/ml) and analyzed the activation of cell survival-related proteins by WB. We observed increased phosphorylation of PDK-1 and Akt only in control cells, confirming that MIF mediates its downstream signaling via CD74 in TNBC cells (Fig. 5c). Overall, these results establish that the downregulation of CD74 in TNBC cells attenuate the autocrine effect of MIF.

### CPSI-1306 induces apoptosis in TNBC cells via regulating the mitochondrial cell death pathway

Induction of ROS and oxidative stress are key factors in inducing apoptotic cell death^29^. Most chemotherapeutic agents are reported to enhance intracellular ROS levels and it is well-accepted fact that the anticancer effect of these chemotherapeutics is mediated by the induction of ROS and oxidative stress^30,31^. In our study, ROS production was measured by H2DCFDA (Sigma-Aldrich, USA). The intracellular ROS-mediated oxidation of DCF-DA to the fluorescent compound 2′,7′-dichlorofluorescein (DCF) was measured by flow cytometry. Following treatment with different doses of CPSI-1306 for 24 h, MDA-MB-468 or MVT-1 cells produced an increased amount of ROS in a dose-dependent manner (Supplementary Fig. 5A).

Intracellular ROS can also hinder mitochondrial functions^32^. Mitochondrial dysfunction is associated with the induction of apoptosis. Depolarization of mitochondrial transmembrane potential (ΔΨ_m_) induces the release of apoptotic factors^33^. To decipher the underlying molecular mechanisms involved in CPSI-1306-mediated apoptosis, we evaluated the mitochondrial membrane potential of CPSI-1306 or vehicle-treated MDA-MB-468 and MVT-1 cells by using tetramethyl rhodamine methyl ester (TMRM) dye^34^. CPSI-1306 treatment caused a decrease in the TMRM-positive signal in viable cells, which indicates the loss of ΔΨm (Supplementary Fig. 5B).

**Fig. 6:**
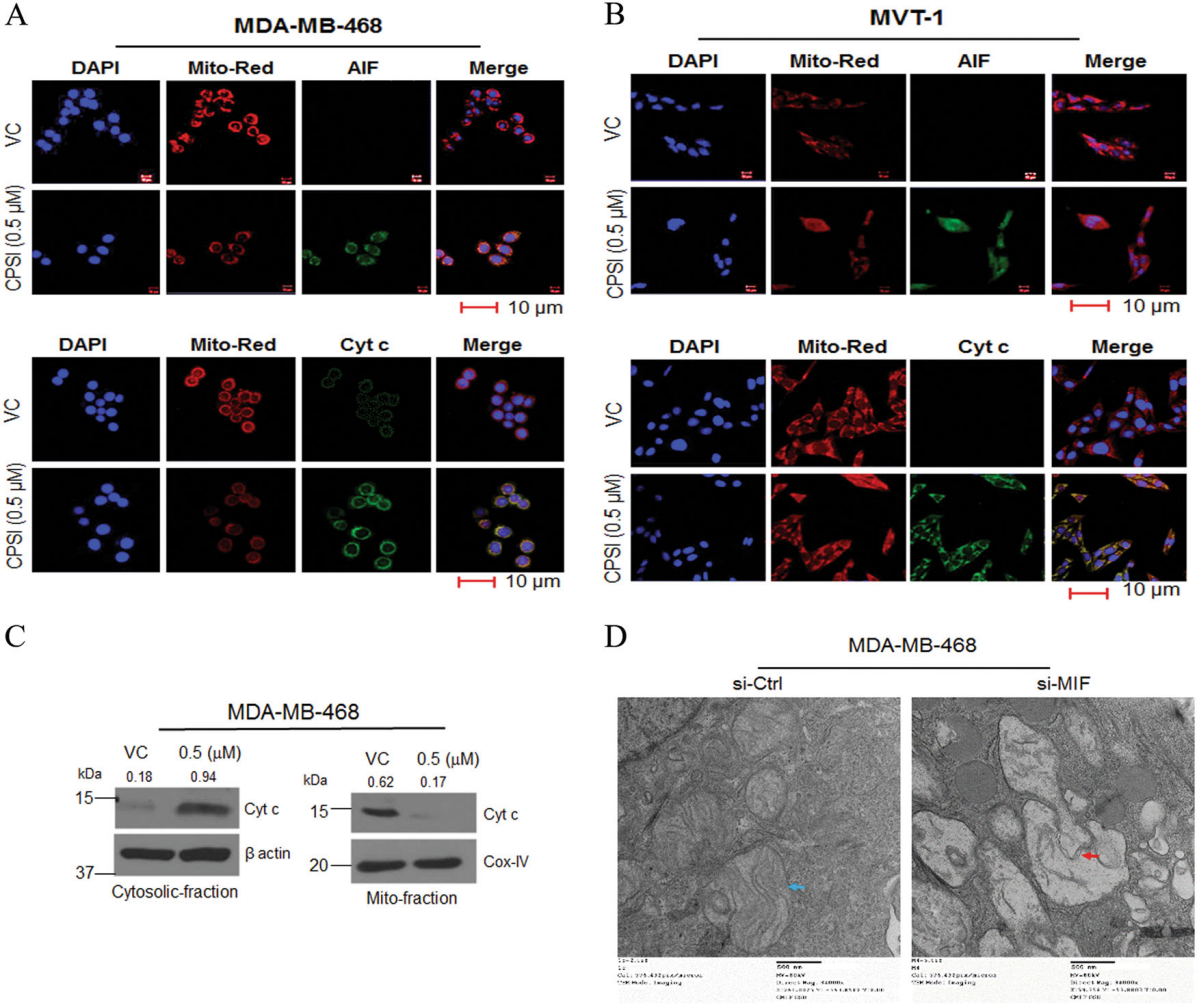
CPSI-1306 treatment induces apoptosis in TNBC cells via the mitochondrial pathway. **a**, **b** TNBC cells (MDA-MB-231 and MVT-1) were seeded in chambered slides and treated with vehicle control (VC) or CPSI-1306 for 48 h followed by immunofluorescence staining with mitochondria selective pro-apoptotic proteins: AIF and Cyt *c* and visualized by confocal microscopy. Mito-Red represent mitochondria. **c** MDA-MB-468 cells were treated with VC or CPSI-1306 (0.5 μM) for 48 h and the release of Cyt *c* was analyzed by WB. Mito: mitochondria. β-Actin and CoxIV served as internal controls. **d** Mitochondrial changes associated with MIF knockdown in MDA-MB-468 cells were revealed by transmission electron microscopy. The red arrow shows mitochondria became aggregated while blue arrow pointed at the normal shape of mitochondria. Data represent mean ± SEM for each experiment repeated three times with similar results. Scale bars: 10 μm. WB was quantified arbitrarily and values were added on top of each image.

Furthermore, apoptosis-related markers were also analyzed following CPSI-1306 treatment in TNBC cells to establish the role of mitochondria-mediated pathway in the activation of apoptosis. Cyt *c* and AIF are inner mitochondrial membrane proteins and get released in the cytosol during mitochondrial permeabilization. The release of Cyt *c* from mitochondria into the cytosol is one of the characteristic features of intrinsic apoptosis^35^. AIF functions as an NADH oxidoreductase in the normal mitochondria and when released in the cytosol causes DNA fragmentation and apoptosis in a caspase-independent manner^36^. Fluorescence microscopy analysis of CPSI-1306 treated TNBC cells demonstrate that CPSI-1306 treatment increased the release of Cyt *c* and expression of AIF^35^ from mitochondria (Fig. 6a, b). This was further confirmed by cell fractionation whereby CPSI-treated cells were fractionated and the cell fractions were analyzed for protein levels of Cyt *c* by western blotting (Fig. 6c). CPSI treatment caused a significant translocation of Cyt *c* from the mitochondria into the cytosol (Fig. 6c). Once the Cyt *c* is released to the cytosol, it can trigger the intrinsic apoptosis pathway which can further activate downstream caspases, such as caspase-9 and caspase-3. Additionally, morphological mitochondrial alterations associated with MIF were revealed by transmission electron microscopy. We observed that MIF downregulation in MDA-MB-231 cells caused mitochondrial morphological changes that can be related to mitochondrial damage-associated apoptosis. MIF knockdown changed the mitochondrial filamentous form to aggregates whereas the control cells retained the normal shape of mitochondria (Fig. 6d). These observations establish that CPSI-1306 treatment induces mitochondrial apoptosis pathway in TNBC cells by increasing intracellular ROS and mitochondrial dysfunction, resulting in a decrease of ΔΨm, which in turn promotes Cyt *c* and AIF release.

### CPSI-1306 is a potent antitumor agent against TNBC growth and metastasis

**Fig. 7:**
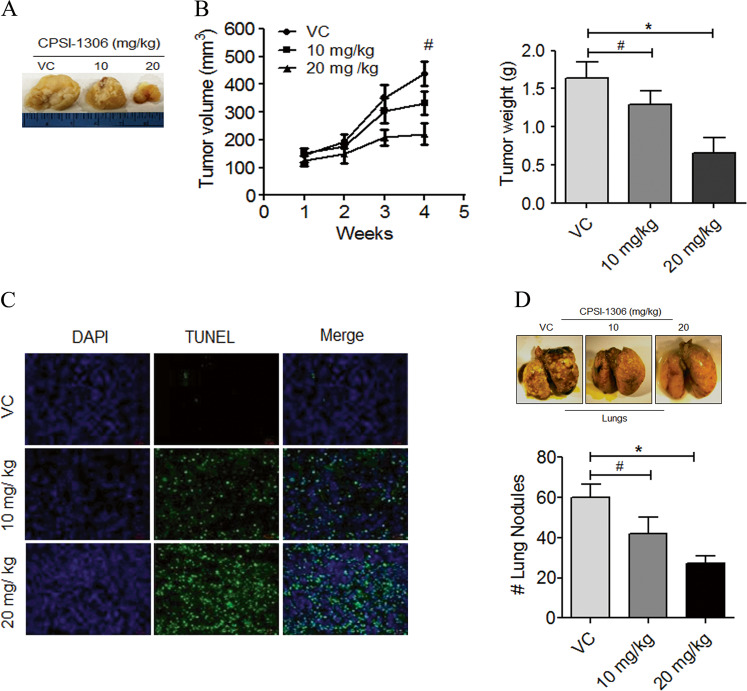
CPSI-1306 inhibits tumor growth, progression, and metastasis in vivo. **a** Briefly, MDA-MB-231 cells (5 × 10^5^) were implanted into the fourth mammary gland of NSG mice (*n* = 5). Once the tumors become palpable, mice were treated with CPSI-1306 (10 mg and 20 mg/kg) or vehicle control (VC) five times a week for up to 4 weeks. Representative tumor images are shown. **b** Tumor volumes (left) were measured externally every week during the CPSI-1306 treatment and tumor weight (right) was calculated at the end of the study. **c** VC or CPSI-1306-treated MDA-MB-231-derived xenograft tumors were analyzed for apoptosis using TUNEL assay **d** Representative photographs of lungs isolated from vehicle or CPSI-1306 treated groups. The bar graph represents the number of metastatic nodules in the lungs. The data reported mean ± SEM of triplicate experiments (*P < 0.05, **P < 0.01, ***P < 0.001, ^#^non-significant).

After observing the strong antitumorigenic activity of CPSI-1306 in vitro, we analyzed its therapeutic potential against TNBC in vivo. The drug safety studies showed that the dose of CPSI-1306 selected for our in vivo studies was well tolerated by animals and did not affect normal organ histology (Supplementary Fig. 6). To explore the antitumorigenic potential of CPSI-1306 in vivo, we utilized human TNBC cell line MDA-MB-231 which has been shown to successfully generate mammary fat-pad xenograft tumors with 100% metastasis rate in NOD/SCID/IL2rγ^null^ (NSG) immunocompromised mice^24^. The cohorts of CPSI-1306 or vehicle control-treated NSG females were evaluated for 4 weeks. The treatment of CPSI-1306 significantly reduced the tumor volume in a dose-dependent manner (Fig. 7a, b). The average weight of CPSI-1306-treated tumors harvested at the end of the experiment were also significantly low compared to vehicle control-treated groups (Fig. 7b).

**Fig. 8:**
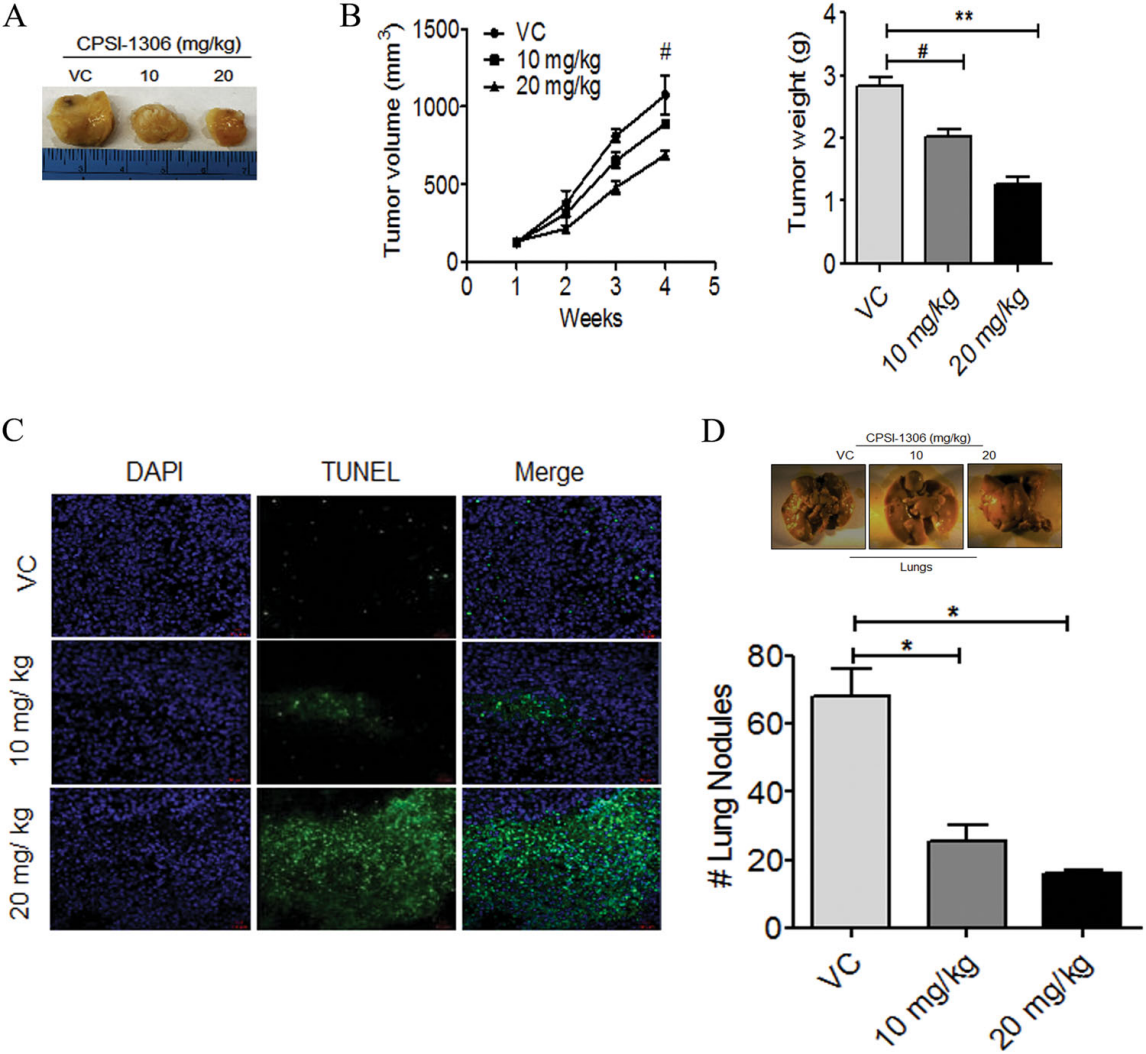
CPSI-1306 inhibits tumor growth, progression, and metastasis in the syngeneic orthotopic mouse model. **a** MVT-1 cells (5 × 10^5^) were implanted into the fourth mammary gland of FVB mice (*n* = 5). Palpable tumors were treated with vehicle control (VC) or CPSI-1306 (10 mg and 20 mg/kg) five times in a week for up to 4 weeks. Representative tumor images are shown. **b** Tumor volumes (left) were measured externally every week during the CPSI-1306 treatment and tumor weight (right) was calculated after the termination of the study. **c** Vehicle or CPSI-1306-treated MVT-1-derived tumors were analyzed for apoptosis using TUNEL assay. **d** Representative photograph of lungs isolated from vehicle or CPSI-1306-treated groups. The bar graph represents the number of metastatic nodules in the lungs. The data reported mean ± SEM of triplicate experiments (*P < 0.05, **P < 0.01, ***P < 0.001, ^#^non-significant).

We further confirm these results using a murine TNBC cell line, MVT-1, derived orthotopic syngeneic tumor model^37^. Similar to MDA-MB-231 tumor data, we found a significant reduction in tumor volume and tumor weight of MVT-1 tumors treated with CPSI-1306 compared to the vehicle control-treated group (Fig. 8a, b). MIF has been shown to increase angiogenesis and dampen antitumor immune surveillance to support tumor cells to acquire metastatic properties^10,25^. IHC analysis of the histological sections derived from CPSI-1306 or vehicle control-treated MDA-MB-231 or MVT-1 tumors were analyzed for the expression of proliferation marker (Ki67) and angiogenic markers (CD31). Our results demonstrate that CPSI-1306 treatment reduced the expression levels of Ki67 and CD31 as compared to vehicle control groups (Supplementary Fig. 7A, B). In addition, expression of VEGF and ICAM-1 were also decreased in these tumors (Supplementary Fig. 8A, B).

To further investigate CPSI-1306-mediated mechanisms of tumor suppression, we analyzed the tumor sections to detect cells undergoing apoptosis through TUNEL assay. The TUNEL assay revealed that the number of apoptotic cells was significantly higher in CPSI-1306-treated MDA-MB-231 or MVT-1-derived tumors than the respective vehicle controls (Figs. 7c and 8c). These results suggest that CPSI-1306 has the potential to inhibit TNBC growth by suppressing tumor cell proliferation, angiogenesis, and inducing apoptosis. These studies illustrate the novel anticancer potency of CPSI-1306 against TNBC.

Next, we examined if CPSI-1306 treatment can also inhibit distant pulmonary metastasis. The MVT-1 and MDA-MB-231 tumor-bearing mice treated with CPSI-1306 or vehicle control were examined for metastatic colonization in the lungs. Gross evaluation of the lungs harvested from mice treated with CPSI-1306 showed a significantly reduced number of lung nodules in a dose-dependent manner (Figs. 7d and 8d). Taken together, these results show that the pharmacological inhibition of MIF using CPSI-1306 inhibits TNBC growth and metastasis in vivo by impeding survival pathways and promoting caspase-dependent apoptotic pathways.

## Discussion

MIF is a multipotent chemokine and a mediator of inflammatory carcinogenesis. Here, we report that MIF expression is elevated in TNBC patients. Although MIF has been shown before to enhance breast tumor growth, its role in TNBC is yet unexplored. Here, for the first time we report that MIF is overexpressed in TNBC using TMAs and publically available datasets. Furthermore, MIF overexpression correlated with worse survival in TNBC compared to other breast cancer subtypes. Recent studies have shown MIF could be used as a biomarker for poor prognosis in several cancers such as glioma, non-small lung cancer, and head and neck squamous cell carcinoma^38,39^.

Our data strongly suggest that MIF plays a key role in tumor growth and metastasis of TNBC. In the present study, we observed MIF-dependent effects on TNBC growth and metastasis using different TNBC cell lines and MIF KO model systems. Orthotopic syngeneic TNBC mammary tumors showed reduced tumor burden in MIF KO mice compared to WT mice. In addition, stable downregulation of MIF in human TNBC cells also resulted in reduced growth and metastasis in vitro and in vivo. Similar to its role in other cancers, we strongly emphasize the role of MIF in promoting tumor growth and metastasis in TNBC.

MIF has been shown to promote breast cancer growth and metastasis^40^. Therefore, we used a small-molecule MIF inhibitor, CPSI-1306, an isoxazoline compound reported to inhibit the oncogenic activity of MIF. As CPSI-1306 is a non-toxic and well-tolerated molecule, its clinical manifestation would be very encouraging. We first evaluated the anticancer activity of CPSI-1306 in vitro and observed a significant inhibitory effect on the viability and colony-forming ability of TNBC cells. Further analysis revealed that CPSI-1306 treatment regulates cell-cycle progression and promotes apoptosis. MIF has been reported to induce apoptosis by inhibiting p53 activation and stabilization^26,41^. In our study, it is plausible that CPSI-1306 mediates its antitumor effects by impeding MIF-dependent negative regulation of apoptosis.

Apoptosis is a complicated process and includes various cellular changes such as alteration in ΔΨm and activation of downstream signaling molecules and protease enzymes including the family of caspase proteins^42,43^. Various apoptosis-related proteins, including pro-apoptotic such as BAD, BIM, and AIF and anti-apoptotic like BCL-XL, are the major contributors in maintaining the ΔΨm^44^. We observed that CPSI-1306 treatment in TNBC cells induces apoptosis via the mitochondrial pathway, by altering the ΔΨm and increasing expression of pro-apoptotic proteins and decreased the expression of anti-apoptotic proteins.

ROS are by-products of cellular metabolism which play a crucial role in normal physiological processes. Dysregulation of ROS generation has been implicated in the development of various inflammatory diseases, including cancers^45,46^. An abrupt increase in cellular ROS can spike changes in ΔΨm that may lead to the generation of mitochondrial ROS known to induce mitochondria-mediated apoptosis^47^. We observed that CPSI-1306 treatment in TNBC cells caused apoptosis by increasing intracellular ROS generation and inhibiting the expression of cell survival and proliferation molecules, including Akt, PDK, and RAF pathways. Thus, our data indicate that CPSI-1306 mediates its antitumor effects by increasing ROS generation and suppressing cell survival and proliferation pathways in TNBC cells. We next evaluated the potential of CPSI-1306 to inhibit TNBC in vivo using orthotopic syngeneic or xenograft mouse models, respectively. We observed that CPSI-1306 treatment significantly inhibits tumor growth and metastasis in both the preclinical TNBC mouse models in a dose-dependent manner. In addition, MIF has been implicated in cell proliferation and angiogenesis in breast cancer^48^. MIF has been reported to enhance the aggressiveness of tumor cells by augmenting the angiogenic potential of various human cancers.

Our results coincide with the previous reports, as we detected a reduced number of Ki67-positive and CD31-positive tumor cells in CPSI-1306-treated groups. The expression of angiogenic and pro-tumor molecules including VEGF and ICAM-1 was also decreased in the CPSI-1306-treated groups indicating the anti-angiogenic potency of CPSI-1306 against TNBC. Tumors can relapse and/or reappear as metastatic disease years after the resection of the primary tumor; our data strongly suggest that MIF also plays a key role in spontaneous lung metastasis of TNBC.

Autophagy has been known to promote TNBC growth, and regarded as a potential therapeutic target^49^. A recent report has shown that autophagy inhibition in TNBC cells lead to enhanced MIF secretion, which further stimulates cell survival^49^. Therefore, MIF inhibition using CPSI-1306 could be a potential therapeutic strategy alone or in combination with classical chemotherapy or autophagy inhibitors for the regression of TNBC growth and metastasis.

This study shows for the first time that MIF plays an important role in regulating TNBC growth and metastasis. Furthermore, our study provides a comprehensive analysis of the antitumor effects of MIF inhibitor CPSI-1306 against TNBC. We have also found that CPSI-1306 inhibits TNBC growth and metastasis by activating apoptosis. Overall, our studies indicate that MIF could be used as a novel therapeutic target against aggressive and metastatic TNBC by using small molecular weight chemical inhibitors of MIF. This could be a promising therapeutic strategy as TNBC currently lacks effective targeted therapies.

## Supplementary information

Supplementary Figure 1

Supplementary Figure 2

Supplementary Figure 3

Supplementary Figure 4

Supplementary Figure 5

Supplementary Figure 6

Supplementary Figure 7

Supplementary Figure 8

Supplementary Figure Legends

Supplementary Table
