# Supplementary figures and images for: Macrophage migration inhibitory factor inhibition as a novel therapeutic approach against triple-negative breast cancer
Source: Cell Death Dis. 2020 Sep 17;11(9):774. doi: 10.1038/s41419-020-02992-y (PMC7498597; doi:10.1038/s41419-020-02992-y)

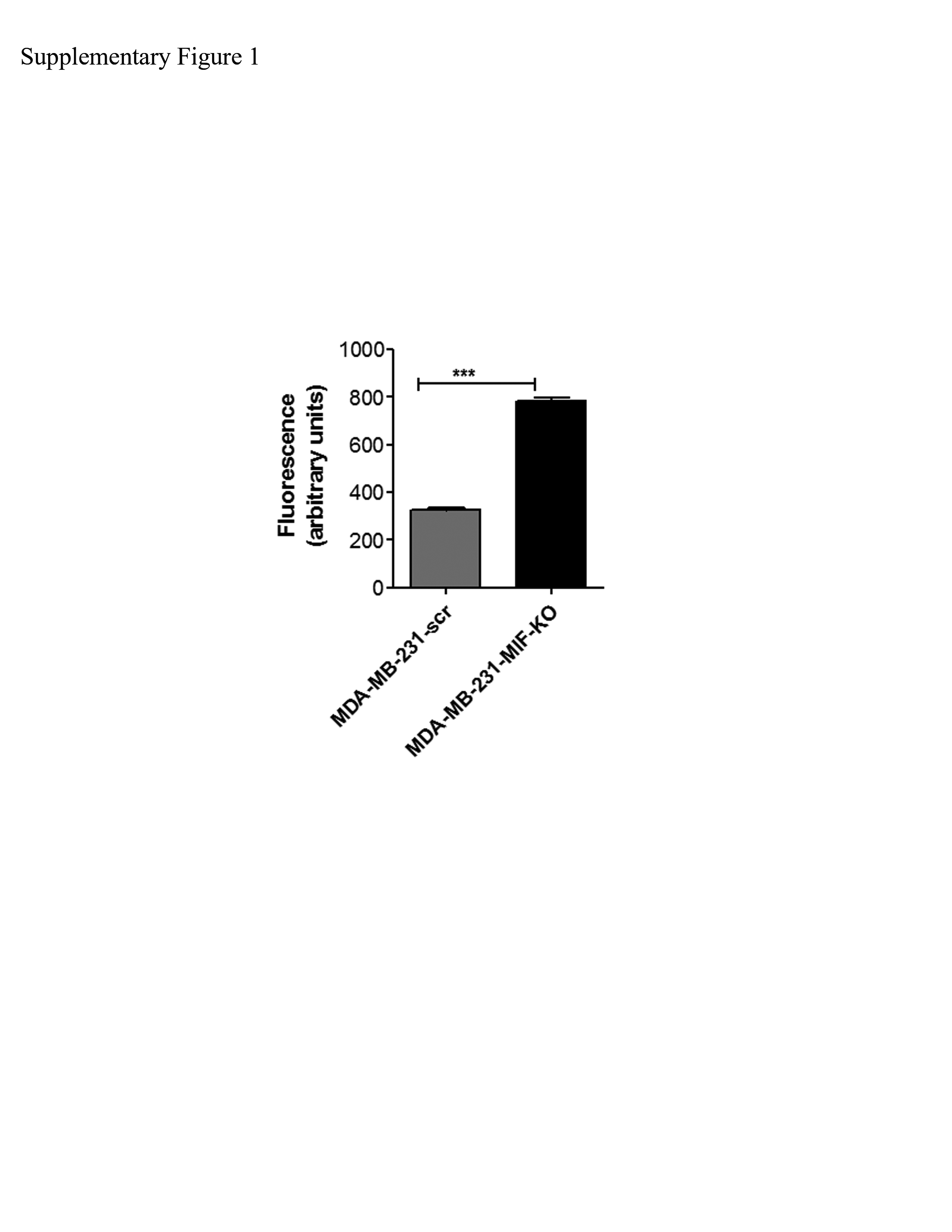

Supplement: Supplementary file 1 — Supplementary Figure 1 [file 41419_2020_2992_MOESM1_ESM.tif]

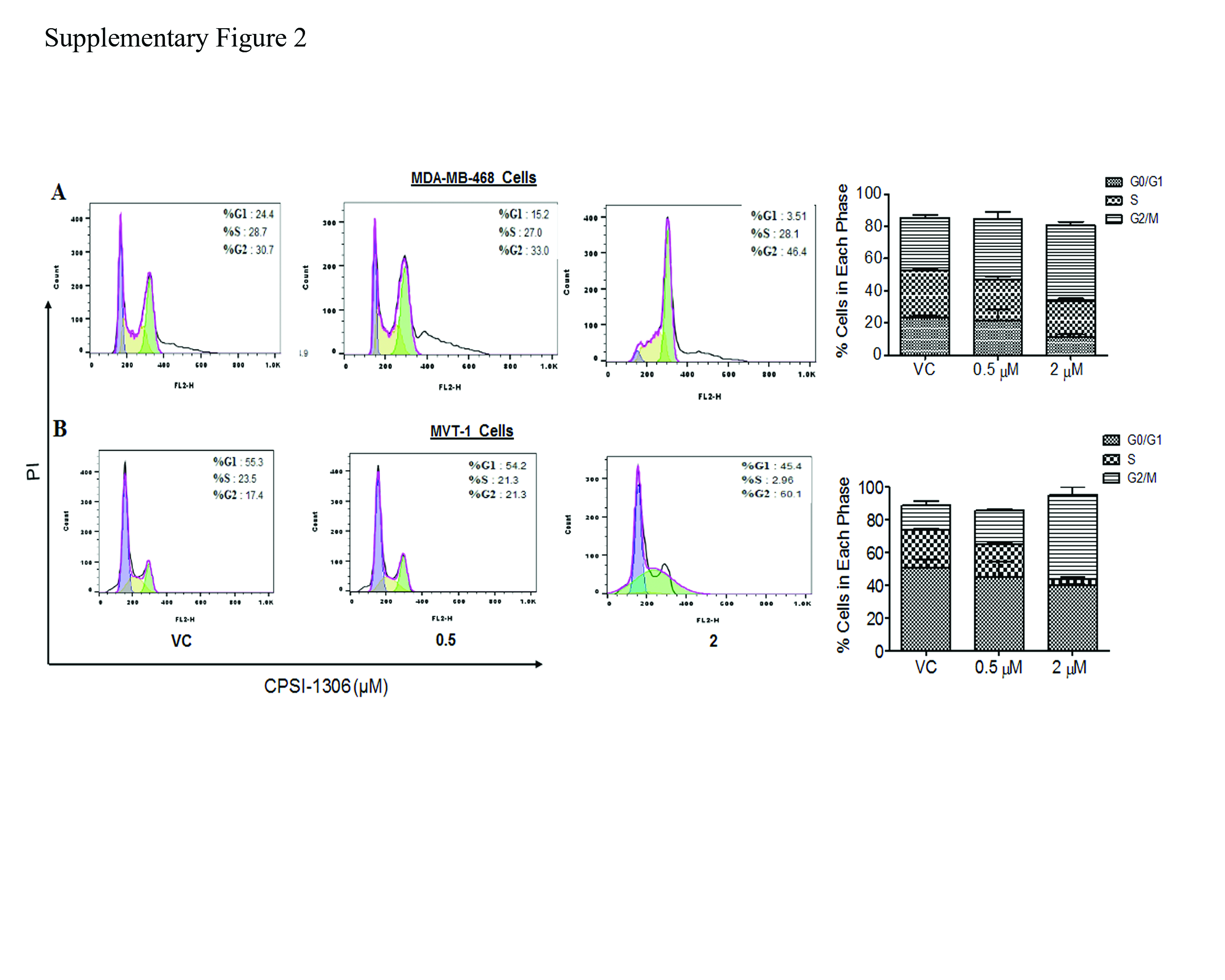

Supplement: Supplementary file 2 — Supplementary Figure 2 [file 41419_2020_2992_MOESM2_ESM.tif]

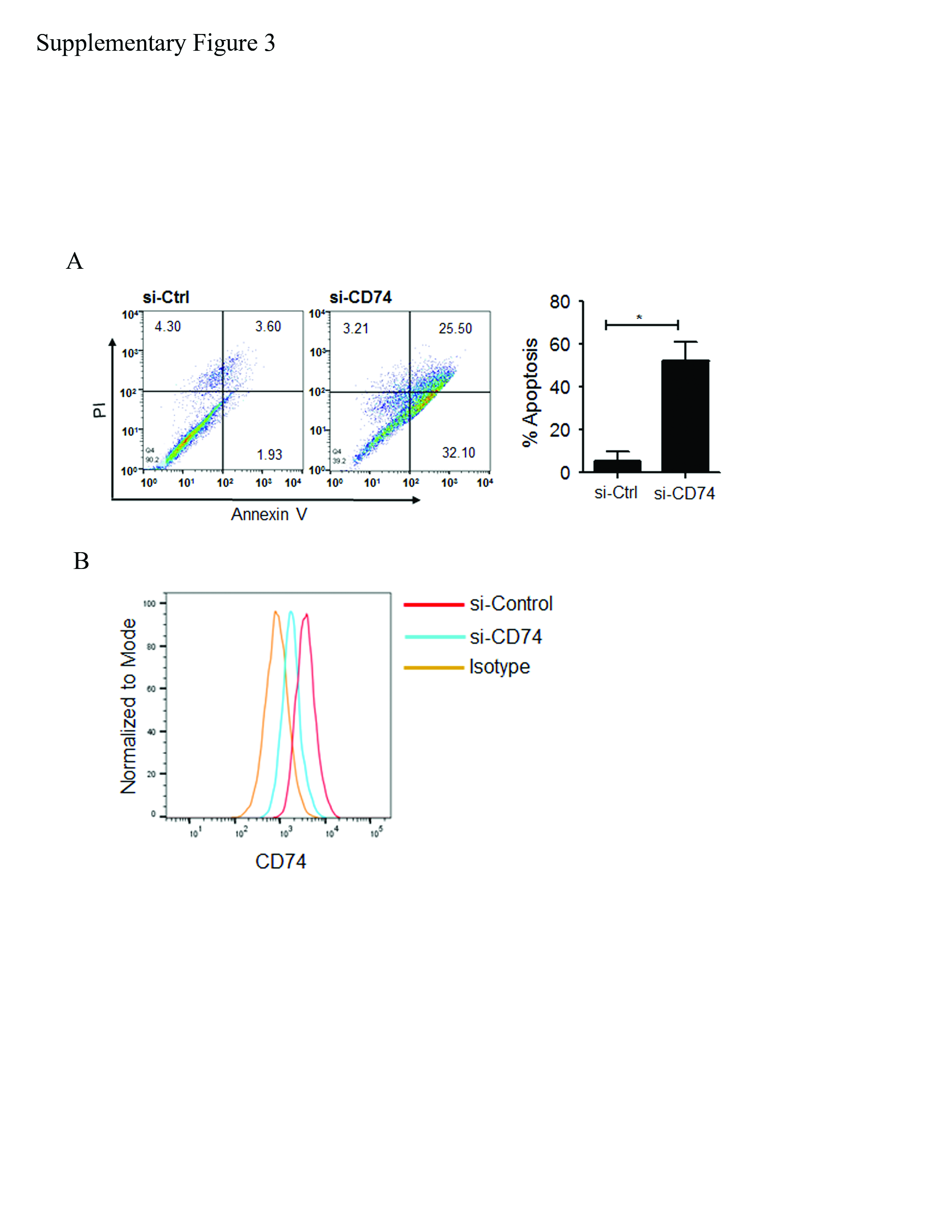

Supplement: Supplementary file 3 — Supplementary Figure 3 [file 41419_2020_2992_MOESM3_ESM.tif]

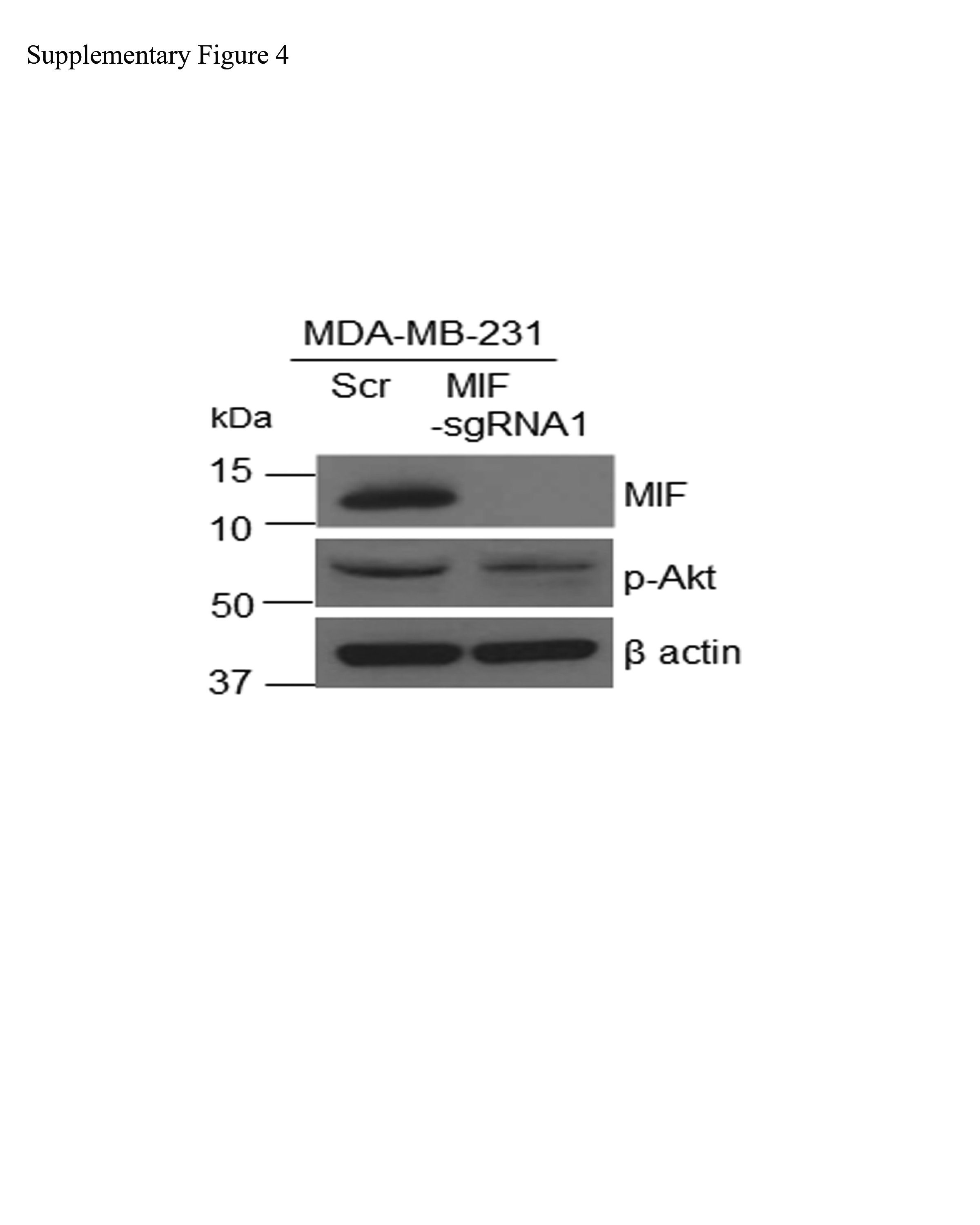

Supplement: Supplementary file 4 — Supplementary Figure 4 [file 41419_2020_2992_MOESM4_ESM.tif]

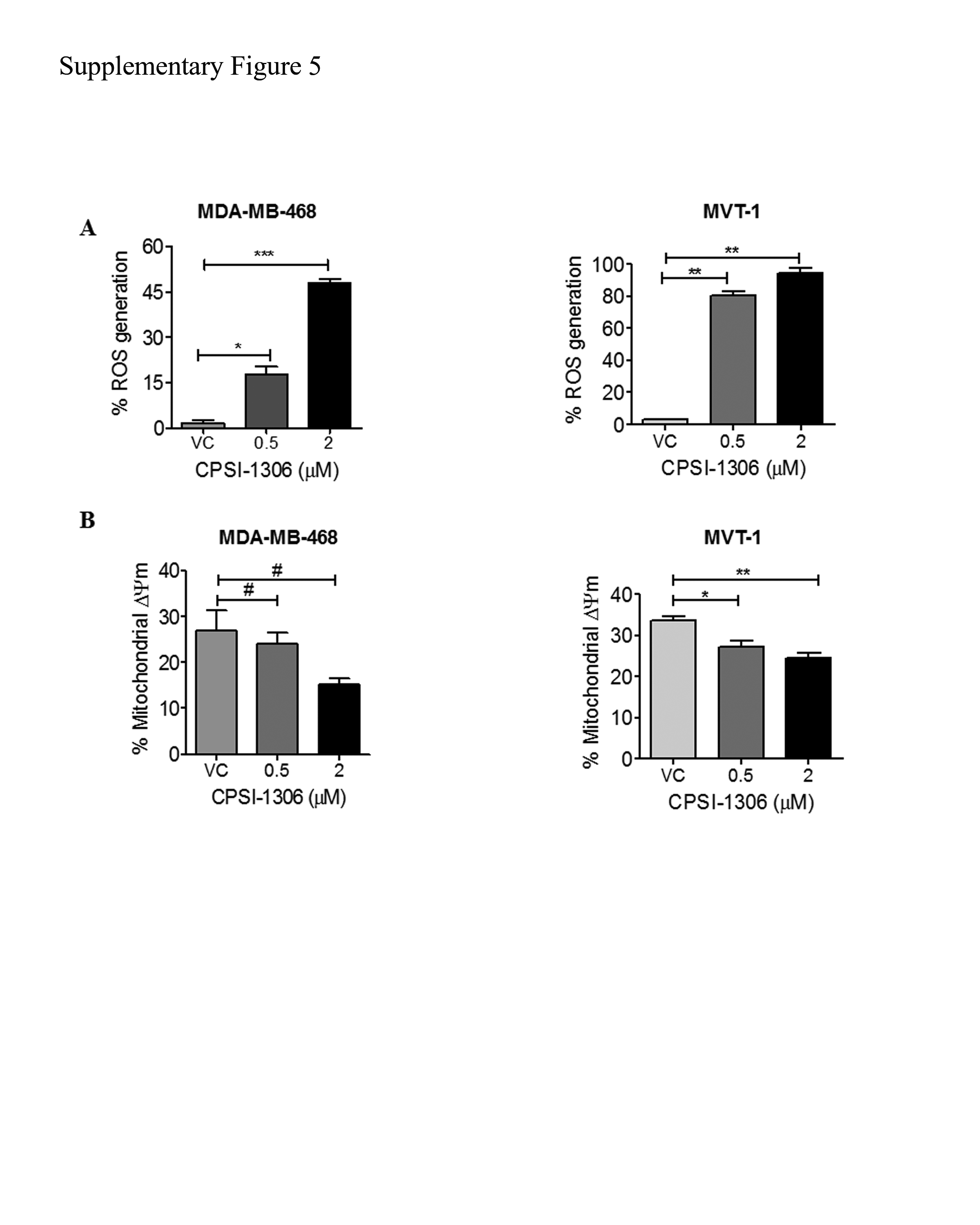

Supplement: Supplementary file 5 — Supplementary Figure 5 [file 41419_2020_2992_MOESM5_ESM.tif]

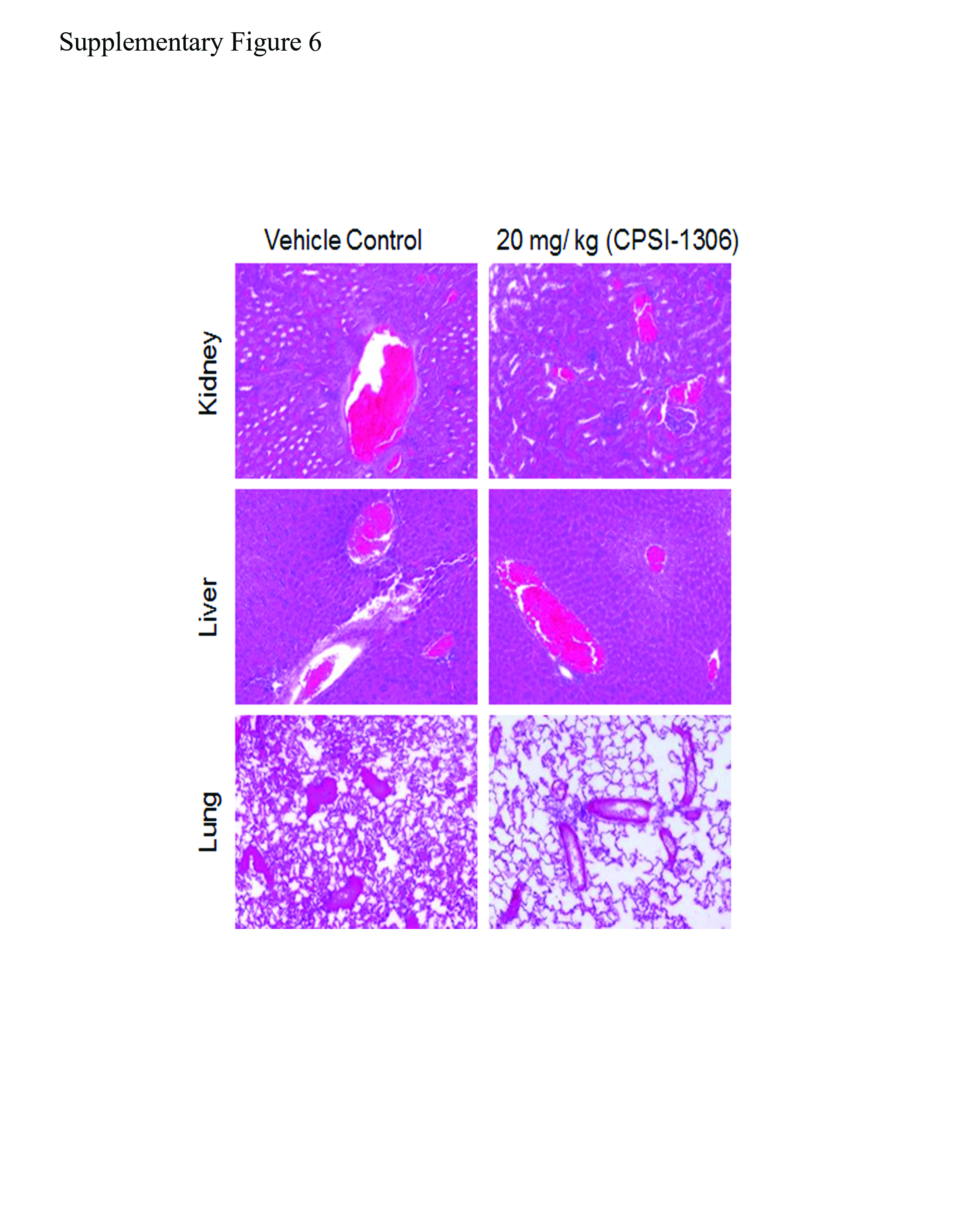

Supplement: Supplementary file 6 — Supplementary Figure 6 [file 41419_2020_2992_MOESM6_ESM.tif]

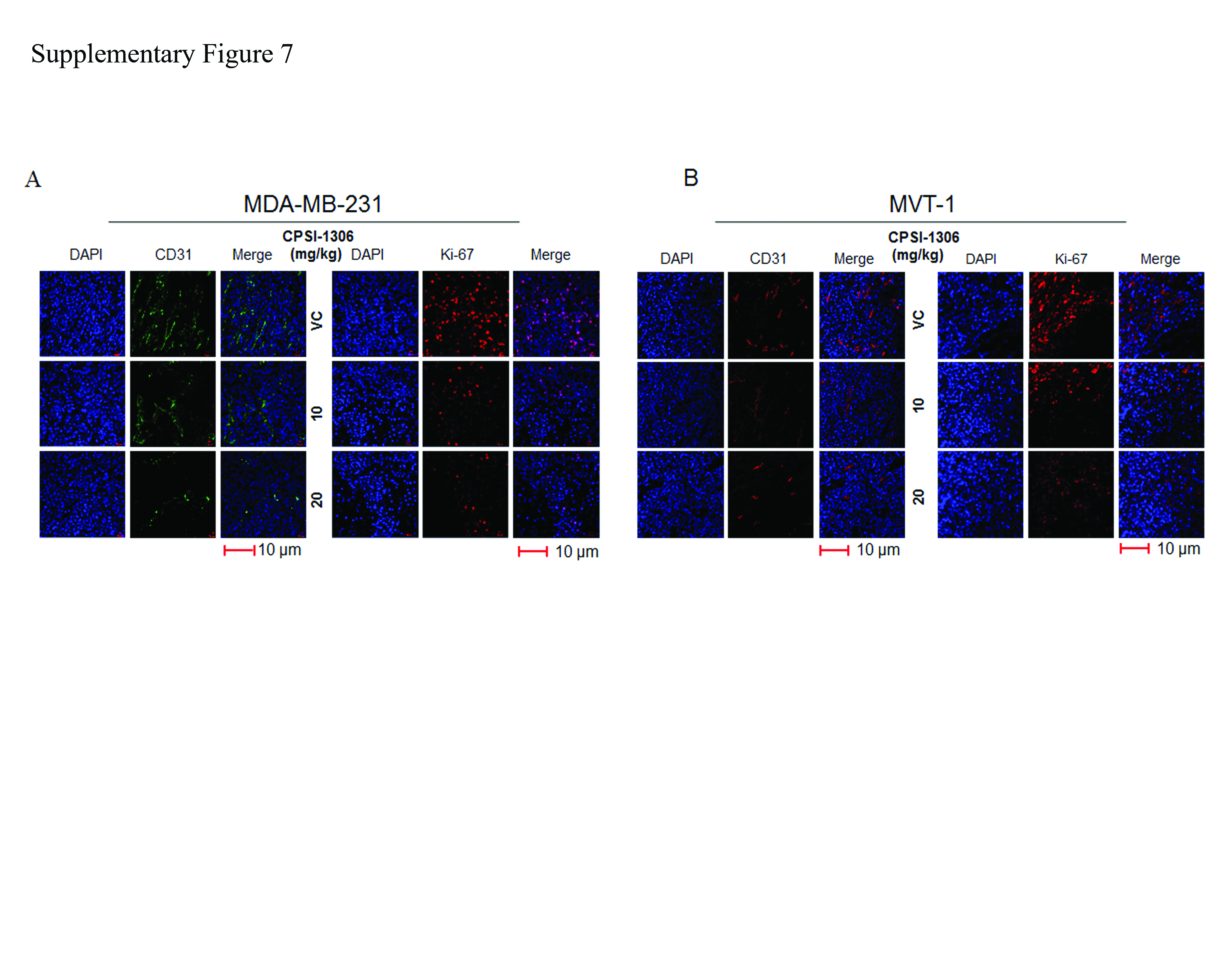

Supplement: Supplementary file 7 — Supplementary Figure 7 [file 41419_2020_2992_MOESM7_ESM.tif]

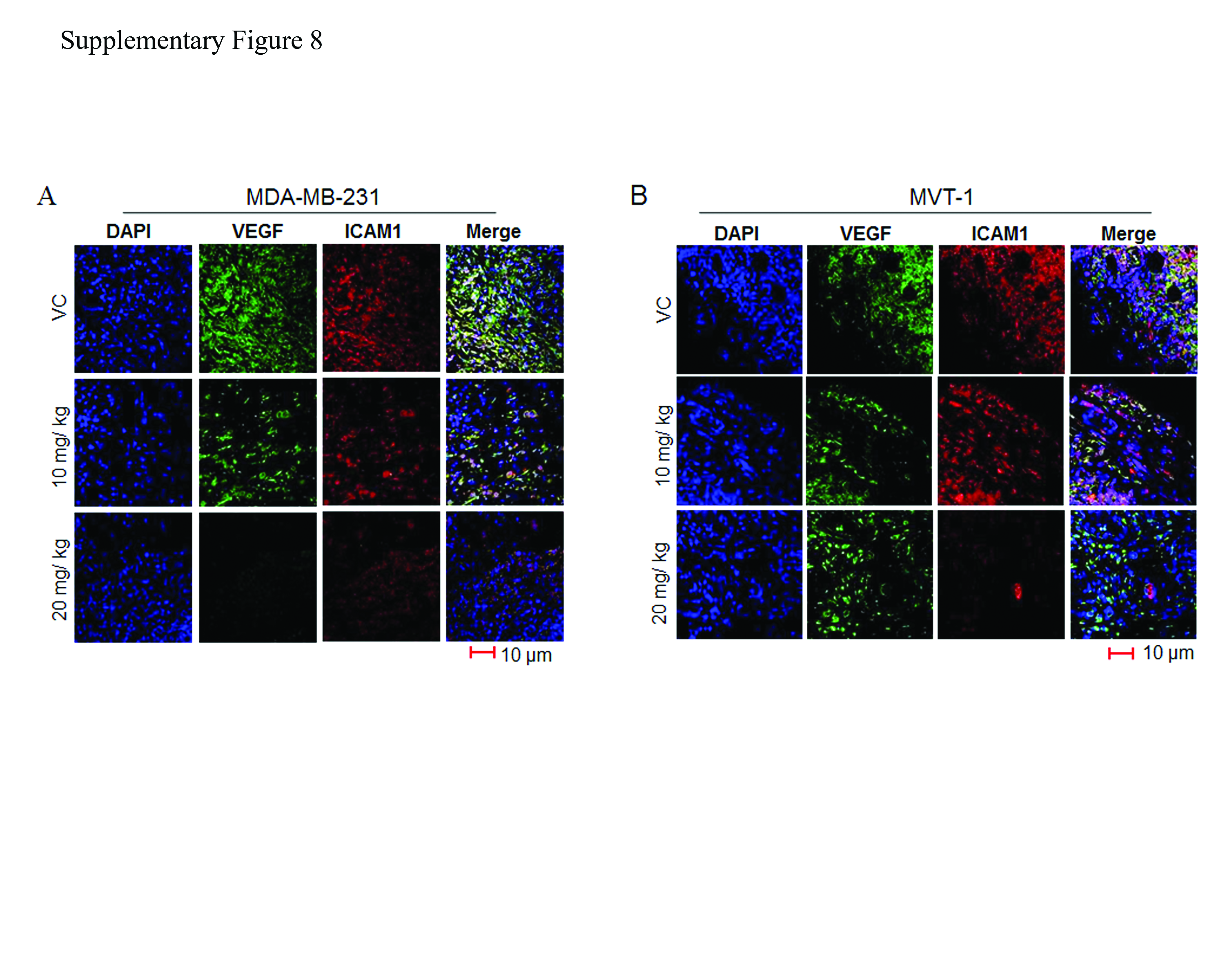

Supplement: Supplementary file 8 — Supplementary Figure 8 [file 41419_2020_2992_MOESM8_ESM.tif]
