# Supplementary material for: Macrophage migration inhibitory factor inhibition as a novel therapeutic approach against triple-negative breast cancer
Source: Cell Death Dis. 2020 Sep 17;11(9):774. doi: 10.1038/s41419-020-02992-y (PMC7498597; doi:10.1038/s41419-020-02992-y)
Supplement: Supplementary file 9 — Supplementary Figure Legends [file 41419_2020_2992_MOESM9_ESM.docx]

**Supplementary figure legends**

**Supplementary Figure 1:** MDA-MB-231 cells were treated with vehicle control (VC) or CPSI-1306 (0.5 μM) and cell viability was analyzed after 48 h using Prestoblue dye. The data reported here is the mean ± SEM of triplicate experiments (*P < 0.05; ** P<0.01).

**Supplementary Figure 2:** Cell cycle analysis was performed using PI in MDA-MB-468 and MVT 1 cells treated with vehicle control (VC) or CPSI-1306 for 48 h. Quantification is in right. The data reported here is the mean ± SEM of triplicate experiments (*P < 0.05; ** P<0.01).

**Supplementary Figure 3:** (A) CD74 was knockdown in in MDA-MB-468 cells using CD74 specific siRNA and percent apoptosis was quantified by Annexin/PI using flow cytometry. (B) CD74 surface expression and its downregulation was analyzed by flow cytometry.

**Supplementary Figure 4:** MIF knockout MDA-MB-231 cells were generated using CRIPR/Cas9 and analyzed for Akt activation**.**

**Supplementary Figure 5:** MDA-MB-468 and MVT-1 cells were treated with vehicle control (VC) or CPSI-1306 and evaluated for (A) reactive oxygen species (ROS) generation and (B) mitochondrial membrane potential using DCFDA and TMRM fluorescent probes, respectively. The data reported as the mean ± SEM of triplicate experiments (*P < 0.05; ** P<0.01, ***P<0.001, # = non-significant).

**Supplementary Figure 6:** H and E staining of the vital organs of mice after treatment with CPSI-1306 for the identification of any toxicity**.**

**Supplementary Figure 7:** Expression of Ki67 and CD31 were determined using IHC in vehicle control (VC) or CPSI-1306 treated tumors derived from (A) MDA-MB-231 and (B) MVT-1.

**Supplementary Figure 8:** Vehicle control (VC) or CPSI-1306 treated (A) MDA-MB-231 and (B) MVT-1 derived tumors were IHC stained for the expression of VEGF and ICAM (N = 3). (C) Western blot analysis of lysates from MDA-MB-231 xenograft tumors for AIF and cleaved caspase 3 expression. GAPDH was used as loading control.
