# Supplementary material for: Macrophage migration inhibitory factor inhibition as a novel therapeutic approach against triple-negative breast cancer
Source: Cell Death Dis. 2020 Sep 17;11(9):774. doi: 10.1038/s41419-020-02992-y (PMC7498597; doi:10.1038/s41419-020-02992-y)
Supplement: Supplementary file 10 — Supplementary Table [file 41419_2020_2992_MOESM10_ESM.docx]

| **Primer name** | **Forward (5’-3’)** | **Reverse (5’-3’)** |
| --- | --- | --- |
| Scramble (scr) | CCGGCCTAAGGTTAAGTCGCCCTCG CTCGAGCGAGGGCGACTTAACCTTAGG TTTTTG | AATTCAAAAACCTAAGGTTAAGTCGCCCTCGCTCGAGCGAGGGCGACTTAACCTTAGG |
| MIF-sh1 | CCGGCAGGGTCTACATCAACTATTACTCGAGTAATAGTTGATGTAGACCCTGTTTTTG | AATTCAAAAACAGGGTCTACATCAACTATTACTCGAGTAATAGTTGATGTAGACCCTG |
| MIF-sh2 | CCGGAACAACTCCACCTTCGCCTAACTCGAGTTAGGCGAAGGTGGAGTTGTTTTTTTG | AATTCAAAAAAACAACTCCACCTTCGCCTAACTCGAGTTAGGCGAAGGTGGAGTTGTT |

**s**hRNA primer sequences:

**Supplementary Table 1**

**1**

**Supplementary Table 2**

| **Cell Line** | **IC-50 (μM)** |
| --- | --- |
| MDA-MB-468 | 0.84 |
| MDA-MB-231 | 1.16 |
| MVT-1 | 0.83 |

**IC_50_** values

| **Antibodies** | **Dilution** | **Company** |
| --- | --- | --- |
| MIF | 1:5000 | Sigma |
| p-Akt (Ser473) | 1:1000 | CST |
| t-Akt | 1:1000 | CST |
| p-PDK1 (Ser241) | 1:1000 | CST |
| Cleaved caspase 3 | 1:1000 | CST |
| Caspase 9 | 1:1000 | CST |
| BCL-XL | 1:1000 | CST |
| Mcl-1 | 1:1000 | CST |
| Cyt c | 1:1000 | CST |
| Cox4 | 1:1000 | CST |
| CD74 | 1:1000 | CST |
| GAPDH | 1:5000 | CST |
| β-actin | 1:10000 | Santa Cruz |

**Supplementary Table 3**
